# ProMENDA: an updated resource for proteomic and metabolomic characterization in depression

**DOI:** 10.1038/s41398-024-02948-2

**Published:** 2024-05-30

**Authors:** Juncai Pu, Yue Yu, Yiyun Liu, Dongfang Wang, Siwen Gui, Xiaogang Zhong, Weiyi Chen, Xiaopeng Chen, Yue Chen, Xiang Chen, Renjie Qiao, Yanyi Jiang, Hanping Zhang, Li Fan, Yi Ren, Xiangyu Chen, Haiyang Wang, Peng Xie

**Affiliations:** 1 Department of Neurology, The First Affiliated Hospital of Chongqing Medical University, Chongqing, 400016 China; 2 NHC Key Laboratory of Diagnosis and Treatment on Brain Functional Diseases, The First Affiliated Hospital of Chongqing Medical University, Chongqing, 400016 China; 3 Department of Health Sciences Research, Mayo Clinic, MN, 55901 USA; 4 The Jinfeng Laboratory, Chongqing, 401336 China; 5 Chongqing Institute for Brain and Intelligence, Chongqing, 400072 China

**Keywords:** Depression, Molecular neuroscience

## Abstract

Depression is a prevalent mental disorder with a complex biological mechanism. Following the rapid development of systems biology technology, a growing number of studies have applied proteomics and metabolomics to explore the molecular profiles of depression. However, a standardized resource facilitating the identification and annotation of the available knowledge from these scattered studies associated with depression is currently lacking. This study presents ProMENDA, an upgraded resource that provides a platform for manual annotation of candidate proteins and metabolites linked to depression. Following the establishment of the protein dataset and the update of the metabolite dataset, the ProMENDA database was developed as a major extension of its initial release. A multi-faceted annotation scheme was employed to provide comprehensive knowledge of the molecules and studies. A new web interface was also developed to improve the user experience. The ProMENDA database now contains 43,366 molecular entries, comprising 20,847 protein entries and 22,519 metabolite entries, which were manually curated from 1370 human, rat, mouse, and non-human primate studies. This represents a significant increase (more than 7-fold) in molecular entries compared to the initial release. To demonstrate the usage of ProMENDA, a case study identifying consistently reported proteins and metabolites in the brains of animal models of depression was presented. Overall, ProMENDA is a comprehensive resource that offers a panoramic view of proteomic and metabolomic knowledge in depression. ProMENDA is freely available at https://menda.cqmu.edu.cn.

## Introduction

Depression is a prevalent mental disorder characterized by low mood and loss of pleasure, with a lifetime prevalence of 11.1%–14.6% [1, 2]. The condition leads to severe functional impairment and was reported as one of the top three leading causes of burden in 2019 [3]. Unlike other somatic diseases, the clinical diagnosis of depression relies entirely on the clinical symptoms of patients, while reliable biomarkers are still lacking [4, 5]. Moreover, the clinical efficacy of antidepressants is limited, with more than one-third of patients demonstrating inadequate treatment responses [6]. Furthermore, the long-term use of antidepressants may lead to various side effects and treatment discontinuation [7]. Therefore, the screening of biomarkers and new drug targets is expected to improve the diagnosis and treatment of depression [8]. Despite substantial research efforts, the precise mechanism underlying the onset of depression remains incompletely defined, and a systematic molecular profile of depression is still lacking.

The rapid development of systems biology technology has led to the emergence of multi-omics approaches as powerful methods enabling the determination of molecular profiles of diseases with complex biological mechanisms [9, 10]. In the field of depression, omics methods, such as metabolomics and proteomics, have been widely applied to study the brain and peripheral samples of patients and animal models. The results have identified numerous differential metabolites and proteins between depressed and normal states [11–13]. Recent developments and applications in omics research have highlighted the need for comprehensive resources that integrate the available knowledge from scattered studies and provide a panoramic view of molecular characterization in depression [14]. Therefore, MENDA was developed to present the manually curated metabolic characterization (with 5,600 metabolite entries) in the context of depression [15]. Since its first release in 2019, multiple integrated studies have employed MENDA to generate meaningful biological insights [16–19]. However, the number of metabolomics studies has doubled since the first release, and the core dataset warrants an update. Moreover, molecular data at the protein level is required to capture the multi-level biochemical dysregulation of depression, which could provide information on specific enzymes in the metabolic pathways and networks [20, 21]. A growing body of proteomics studies have identified alterations in protein abundances in depression [22–24], providing an important resource of proteomic information. To date, a platform facilitating the systematic curation of proteomic changes in depression is still lacking. Adding protein information to the MENDA will contribute to a deeper understanding of molecular alterations in depression.

This study aimed to create a standardized resource for all available knowledge in the growing area of proteomic and metabolomic research in depression. Therefore, this study presents Protein and Metabolite Network of Depression Database (ProMENDA), an upgraded resource that provides a platform for the manual annotation of candidate proteins and metabolites linked to depression. The data set now contains 43,366 molecular entries from 1370 studies that investigated differential proteins and metabolites in both patients and animal models of depression. This update represents a significant increase (more than 7-fold) in molecular entries compared to the initial release of MENDA. Additionally, the web interface was redesigned to enhance the ease of use. The design and implementation of these updates and changes are described below. To demonstrate the usage of ProMENDA, a case study analyzing the molecular changes in the brains of animal models is presented. ProMENDA is expected to contribute to the investigation of the molecular profile of depression.

## Materials and methods

### Overview of ProMENDA framework

**Fig. 1:**
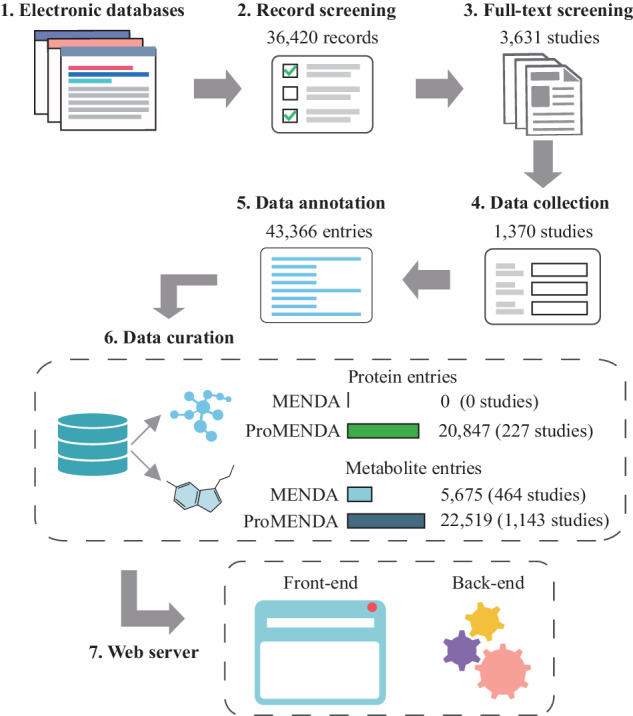
The workflow of the study. The flowchart of the construction process for the ProMENDA.

The schematic overview of ProMENDA is illustrated in Fig. 1. In brief, information on study design and candidate molecules from proteomics and metabolomics studies of depression was manually collected using standardized data extraction tables. These tables were then integrated into annotated datasets based on a multi-faceted annotation scheme. In addition, a brand-new web interface was developed in this update to enhance user experience.

### Creation of the proteomic dataset

Data from proteomic studies of depression were incorporated to expand the scope of MENDA. Relevant proteomic studies were searched from PubMed, Embase, Web of Science, and PsychInfo (Table S1), resulting in a cumulative total of 13,742 literature records retrieved as of May 18, 2023 (Table S2). Studies that investigated proteomic changes associated with depression and its treatment in both human and animal models were screened based on titles and abstracts, yielding 436 potential studies. After checking the full text of these studies, 227 studies were finally included. The exclusion reasons are summarized in Table S3.

Subsequently, a proteomic dataset was created by manually selecting the study information and differentially expressed proteins from the full texts and supplementary materials of these studies using a standardized data abstraction spreadsheet (Table S4). To present experimental information on each protein, the proteomic data were annotated based on a multi-faceted annotation scheme as described in MENDA [15], with minor modifications. The annotation scheme involved manual annotation of information of interest at both the study and protein levels, including experimental design, types of organisms, categories of depression, categories of tissues, experimental techniques, citations, etc.

Moreover, protein information was annotated to ensure standardization, including UniProt accessions, UniProt entry names, protein names, and gene symbols based on the UniProtKB database (version 2023_03) [25]. This step was necessary as proteins were presented in different formats in the original reports, such as protein names, Uniprot accession, or gene symbols. A total of 20,847 protein entries were finally curated.

### Update for metabolomic dataset

In this update, MENDA was expanded to include more comprehensive and up-to-date metabolite entries. The initial release of MENDA included 5675 metabolite entries from 464 metabolomics and magnetic resonance spectroscopy studies as of March 20, 2018. To ensure that the ProMENDA remains current, literature databases were re-screened as of May 18, 2023, resulting in a cumulative total of 22,678 records. After screening the full text of 3195 studies, a total of 1143 eligible studies were included in ProMENDA. The reasons for the exclusion of the ineligible studies are summarized in Table S3. Using a similar data extraction and annotation process to the proteomic dataset, a total of 22,519 differential metabolite entries were curated in this update. The expansion of the metabolite dataset will provide researchers with a more comprehensive overview of metabolic changes associated with depression.

### Web interface implementation

The front end of the ProMENDA website has been implemented using HTML, JavaScript, and CSS. The web interface was designed using Bootstrap (https://getbootstrap.com/) and jQuery (https://jquery.com/) libraries, and the interactive tables were constructed using the DataTables library (https://datatables.net/). In addition, the website is hosted on an Apache server (https://httpd.apache.org).

### Case study for applications of ProMENDA

In addition to data browsing, users can download data from ProMENDA to conduct data mining studies. To illustrate the potential applications of ProMENDA, a case study involving an integrated analysis was conducted based on protein and metabolite entries in the brains of animal models of depression. Metabolite and protein entries were selected based on the following criteria: (1) differential molecules between depressed vs. healthy states; (2) all types of animal models; (3) all brain tissues; and (4) all analytical platforms. Semi-quantitative analyses were performed based on selected data. Considering the possibility of inconsistencies in the up- and down-regulation of certain molecules (with unique gene symbols or metabolite names) across different studies, a vote-counting method was utilized for semi-quantitative analysis. This approach effectively identifies the molecules exhibiting consistent up- or down-regulation under specific conditions, indicating their high reproducibility and potential as biomarkers [26]. Furthermore, the *binom.test* function in R (version 4.3.0, https://www.rproject.org/) was used based on the downloaded data of interest to identify consistently differentially expressed molecules [27]. Candidate molecules with one-tailed *P* < 0.05 across different studies were considered as consistently altered. ImageGP was used to create plots [28].

## Results

### Data summary

The initial release of MENDA comprised 5675 metabolite entries. In ProMENDA, significant efforts have been made to expand the molecular entries. A cumulative total of 36,420 records were screened from electronic databases, and after checking the full texts of 3631 studies, 1370 studies that investigated the levels of proteins and metabolites in depression and its treatment were included. A standardized data extraction and annotation process was adopted, and 43,366 molecular entries were curated, including 20,847 protein entries (Supplementary Data 1) and 22,519 metabolite entries (Supplementary Data 2). This has resulted in a significant increase (more than 7-fold) in molecular entries compared to the previous version of MENDA (Fig. 1). Moreover, our laboratory provided 3173 metabolites entries and 3927 protein entries in ProMENDA, accounting for 16.4% of all molecular entries.

**Fig. 2:**
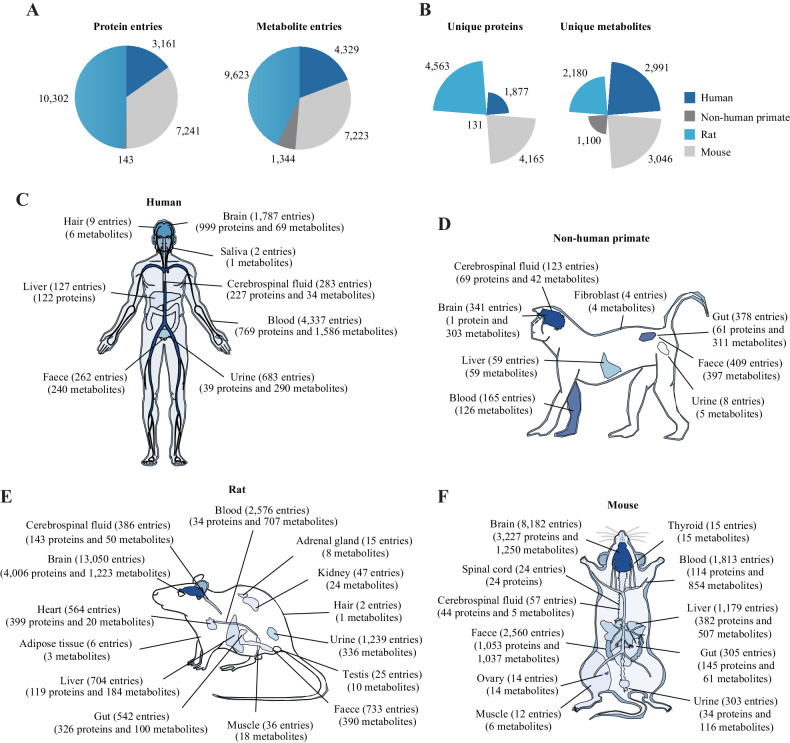
An overview of the included studies and molecular entries from human, non-human primate, rat, and mouse studies. **A** The number of molecular entries from each organism. **B** The number of unique proteins and metabolites from each organism. **C** The number of molecular entries and unique molecules in 8 human tissues. **D** The number of molecular entries and unique molecules in 8 non-human primate tissues. **E** The number of molecular entries and unique molecules in 14 rat tissues. **F** The numbers of molecular entries and unique molecules in 11 mouse tissues.

The number of studies that explored molecular alterations in each organism and each tissue is shown in Fig. S1. In ProMENDA, 7490 molecular entries were curated from human tissues, 1487 from non-human primate tissues, 19,925 from rat tissues, and 14,464 from mice (Fig. 2A). The numbers of unique proteins and metabolites in humans, non-human primates, rats, and mice are shown in Fig. 2B. Specifically, in humans, 3161 protein entries (from 1877 unique proteins) and 4329 metabolite entries (from 2991 unique metabolites) were curated from 8 types of tissues (Fig. 2C). In non-human primates, 143 protein entries (from 131 unique proteins) and 1344 metabolite entries (from 1100 unique metabolites) were curated from 8 types of tissues (Fig. 2D). In rats, 10,302 protein entries (from 4563 unique proteins) and 9623 metabolite entries (from 2180 unique metabolites) were collected from 14 types of tissues (Fig. 2E). In mice, 7241 protein entries (from 4165 unique proteins) and 7223 metabolite entries (from 3046 unique metabolites) were obtained from 11 types of tissues (Fig. 2F). The most frequently reported proteins and metabolites in each organism are displayed in Fig. S2. Among these molecular entries, 72.7% of protein entries and 63.8% of metabolite entries were collected from studies that compared molecular levels between depressed and healthy states; the remaining entries were collected from studies that investigated molecular changes resulting from antidepressant treatments.

### Web interface of ProMENDA

To facilitate the storage and access of study-level and molecular-level datasets, a new web interface was developed for ProMENDA. This interface comprises three main web pages, including Browse, Search, and Download.

**Fig. 3:**
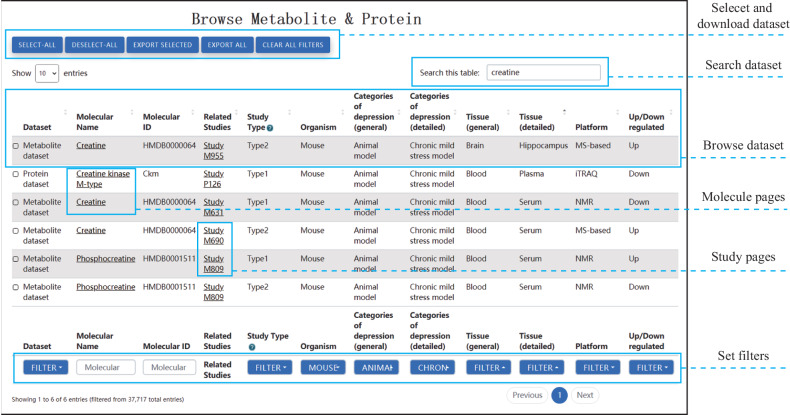
The user-friendly web interface of ProMENDA. The web interface allows users to easily browse, search, filter, and download molecular entries of interest. The browse page displays molecular entries in an interactive table format, enabling users to quickly access relevant information.
(ii) (ii) Search page: On the Search page, users can search for protein or metabolite entries using molecular names or IDs. Each query generates hyperlinks for relevant molecules and their links to other databases, including UniProt, HMDB, KEGG, and PubChem [25, 29–31].
(iii) (iii) Download page: The Download page provides free access to the core datasets of ProMENDA, with additional information available in the Excel documents.
(iv) (iv) Others: In addition to the pages mentioned above, ProMENDA also offers other pages in its web interface, including Home, Introduction, News, Tutorial, and Contact pages. These pages enhance the user experience and provide users with a complete understanding of ProMENDA and its features.

(i) Browse page: The Browse page features interactive tables that present molecular and study information, providing web links for each study and molecule (Fig. 3). Users can easily search these tables by using search columns and applying filters based on study type, organism, categories of depression, tissue, platform, and up/down-regulation. Users are also allowed to select and download data of interest. Basic information about the molecule is provided on the web page of each molecule and the related data entries are summarized. On the web page of each study, its experimental design and candidate molecules are listed.

### Use case: investigating molecular changes in the brains of animal models of depression

**Fig. 4:**
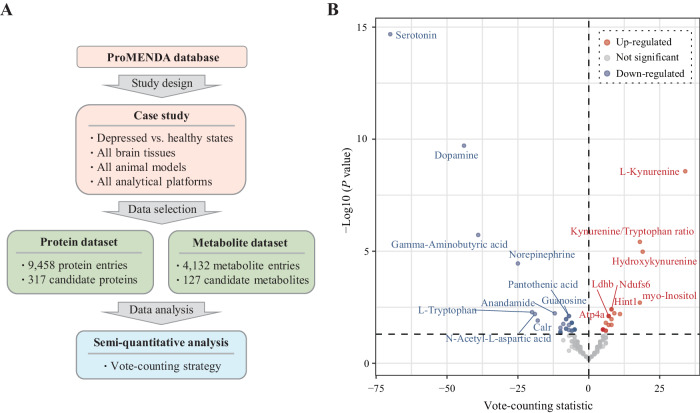
The comprehensive analysis results for the case study. **A** Overview of the study design for the case study. **B** Volcano plot showing consistently dysregulated molecules in the vote-counting procedure.

To illustrate the usage of ProMENDA, a case study was conducted to investigate the molecular changes of candidate proteins and metabolites in the brains of animal models of depression (Fig. 4A). Based on the inclusion criteria, 9458 protein entries and 4132 metabolite entries were initially included from the ProMENDA database. From these molecular entries, 317 candidate proteins and 127 candidate metabolites (or metabolite ratios) were identified from more than four studies. The results of the vote-counting strategy showed that the levels of 28 proteins and 32 metabolites were consistently dysregulated in the brain (*P* < 0.05; Fig. 4B and Table S5).

## Discussion

Depression is a prevalent disease with complex molecular alterations, and exploring potential molecular changes presents an opportunity to unravel the neurobiology and treatment targets of depression [32]. In recent years, numerous clinical and preclinical studies with various designs have employed high-throughput assays to study the molecular changes associated with depression. However, collecting and integrating this massive amount of data from a systems biology perspective remains challenging. Therefore, MENDA was previously developed, containing over 5000 metabolite entries associated with depression. Building on this foundation, ProMENDA was created, presenting a significant extension of the initial release of MENDA, which includes both a new protein dataset and an updated metabolite dataset.

The prime objective of this study was to create a comprehensive knowledge base for proteomic and metabolomic characterization in depression, which may serve as a valuable resource for researchers in the field of depression. To complement the initial release of MENDA, the molecular entries were expanded in ProMENDA by adding a proteomic dataset. While metabolic disturbance only covers a small portion of all biological processes in depression, a platform facilitating the systematic curation of molecules other than metabolites is required. Proteins are major macromolecules that are involved in complex biological functions and regulate metabolic processes; hence, proteomics is a powerful technology enabling a comprehensive understanding of human biology [33–35]. To provide a deeper understanding of candidate protein alterations in depression, 20,847 protein entries were manually curated from 227 studies, which significantly expanded our knowledge base of proteomic alterations in depression. ProMENDA demonstrates the molecular landscape of depression at both the protein and metabolite levels. Compared to Pharos, which contains about 1318 candidate proteins associated with depression based on evidence from proteins, genes, or transcriptions [36], ProMENDA provides a significantly wider selection of protein entries and rich annotation information.

In addition to expanding the proteomic dataset, the metabolite dataset was also updated in ProMENDA. Currently, 22,519 metabolite entries are provided, which is more than three times the number of entries since the initial release of MENDA. The metabolite entries in ProMENDA were compared with other knowledge bases such as MetSigDis, which provides a comprehensive resource of metabolite alterations in 129 diseases [37]. Compared to MetSigDis, ProMENDA provided a 3.2-fold higher number of metabolite entries for pan-diseases and a 600-fold higher number of metabolite entries for depression. This significantly higher number of metabolite entries in ProMENDA will provide researchers with a more comprehensive understanding of the metabolomic landscape of depression. Additionally, a new web interface was implemented in this update, integrating data browsing, searching, selecting, filtering, and downloading functions, improving user access to data analysis.

The efficient usage of ProMENDA was demonstrated in a case study investigating molecular alterations in the brains of animal models of depression. Compared to the initial release of MENDA, which only contained metabolite data, the integrated analysis based on changes in both protein expression and metabolite concentrations provided a more comprehensive insight due to the chemical and functional diversities between proteins and metabolites, as well as their interactions [38]. The results of the vote-counting strategy in the case study revealed that 60 molecules were consistently up-regulated or down-regulated across animal studies. Expectedly, this case study found significantly decreased levels of several neurotransmitters, including serotonin, dopamine, gamma-aminobutyric acid, and norepinephrine, which further support the role of monoamines and gamma-aminobutyric acid in depression [39, 40]. In addition to the analysis methods mentioned in the case study, users are also encouraged to perform further analysis using other analytical strategies and analysis tools. In a previous study, Fu et al. constructed a comprehensive knowledge graph focusing on food, gut microbiota, and mental diseases, which incorporated metabolite-disease associations from our database [41]. Moreover, Gao et al. performed a comprehensive analysis of metabolites in the hippocampus of depression models based on our database. They employed pathway analysis and experiment validation and found that disturbances in the neurotransmitter pathways of the hippocampus were associated with depression [42]. However, users should take data heterogeneity into account during study design, such as differences between human and animal studies, the subtypes of depression, and the type of tissues.

## Limitations

Despite the significant improvements made in ProMENDA compared to MENDA, the platform still has some limitations. Only data from omics studies was included, so molecular entries from traditional experimental methods such as Western blotting have not been included. The slow manual curation process poses a major challenge for researchers who wish to collect and annotate molecular entries from all relevant publications [43]. However, the continuous progress of biomedical text mining techniques might facilitate the curation of large-scale molecular data from scattered literature [44, 45]. Another limitation of ProMENDA is the need for additional omics data, such as genomic and transcriptomic data, reflecting the multifactorial molecular changes that occur in depression. Data curation for the ProMENDA database will be an ongoing process. ProMENDA provides a more comprehensive understanding of the molecular landscape of depression; subsequent updates will facilitate the development of new therapeutic strategies.

## Conclusions

In summary, ProMENDA is a valuable resource for depression research, offering a significant expansion of the core dataset compared to the initial release of MENDA. The current version of ProMENDA includes 43,366 molecular entries, comprising 20,847 protein entries and 22,519 metabolite entries, which were manually curated from 1370 human, rat, mouse, and non-human primate studies. This represents a more than seven-fold increase in molecular entries compared to the previous version of MENDA. ProMENDA is freely accessible to the public at https://menda.cqmu.edu.cn and provides a new user-friendly web interface that allows users to browse, search, select, filter, and download data.

### Supplementary information

Supplement

Dataset 1

Dataset 2

## Data Availability

The datasets generated in the current study are available in Supplementary Data 1 and Supplementary Data 2, or on the ProMENDA website (https://menda.cqmu.edu.cn).
