# Supplementary material for: ProMENDA: an updated resource for proteomic and metabolomic characterization in depression
Source: Transl Psychiatry. 2024 May 30;14:229. doi: 10.1038/s41398-024-02948-2 (PMC11139925; doi:10.1038/s41398-024-02948-2)

**Supplementary information**

**ProMENDA: an updated resource for proteomic and metabolomic characterization in depression**

Pu *et al*. Translational Psychiatry.

**Supplemental Tables ...................................................................................................................... 2**

Table S1. Key terms for search of electronic databases (proteomics data) ................................ 2

Table S2. Numbers of potential citations for proteomics studies ............................................... 4

Table S3. Numbers of full-text articles excluded ........................................................................ 5

Table S4. An example of the standardized data abstraction spreadsheet .................................... 6

Table S5. Consistently altered molecules identified by vote-counting procedure ...................... 9

**Supplemental Figures ................................................................................................................... 13**

Fig. S1. An overview of included studies and molecular entries in human, non-human primate, rat, and mouse studies. .............................................................................................................. 13

Fig. S2. The number of entries for the most frequently reported molecules in human, non-human primate, rat, and mouse studies. .................................................................................................14

**Supplemental Dataset 1. The core dataset for proteomic data.**

**Supplemental Dataset 2. The core dataset for metabolomic data.**

Supplemental Tables

| **Table S1. Key terms for search of electronic databases (proteomics data).** | |
| --- | --- |
| **Key terms for search of PubMed** | |
| #1 | Proteome[MeSH Terms] OR Proteomics[MeSH Terms] OR Mass Spectrometry[MeSH Terms] OR Electrophoresis, Gel, Two-Dimensional[MeSH Terms] OR proteom*[Title/Abstract] OR mass spectrometry[Title/Abstract] OR mass-spectrometry[Title/Abstract] OR mass spectrometric[Title/Abstract] OR isobaric tags for relative and absolute quantification[Title/Abstract] OR iTRAQ[Title/Abstract] OR Tandem Mass Tag[Title/Abstract] |
| #2 | depressive disorder[MeSH Terms] OR depress*[Title/Abstract] |
| #3 | #1 AND #2 |
| **Key terms for search of Embase** | |
| #1 | proteom* OR 'mass spectrometry' OR 'mass-spectrometry' OR 'mass spectrometric' OR 'isobaric tags for relative and absolute quantification' OR iTRAQ OR 'Tandem Mass Tag':ti,ab |
| #2 | Proteome/exp OR Proteomics/exp OR 'Mass Spectrometry'/exp OR 'Electrophoresis, Gel, Two-Dimensional'/exp |
| #3 | #1 OR #2 |
| #4 | depress*:ti,ab |
| #5 | 'depressive disorder'/exp |
| #6 | #4 OR #5 |
| #7 | #3 AND #6 |
| #8 | #7 AND ('article'/it OR 'article in press'/it OR 'chapter'/it OR 'letter'/it OR 'review'/it OR 'short survey'/it) |
| **Key terms for search of Web of Science** | |
| #1 | TS=(proteom* OR “mass spectrometry” OR “mass-spectrometry” OR “mass spectrometric” OR “isobaric tags for relative and absolute quantification” OR iTRAQ OR “Tandem Mass Tag”) |
| #2 | TS=(depress*) |
| #3 | #1 AND #2 |
| **Key terms for search of PsycINFO** | |
| #1 | (proteom* OR "mass spectrometry" OR "mass-spectrometry" OR "mass spectrometric" OR "isobaric tags for relative and absolute quantification" OR iTRAQ OR "Tandem Mass Tag").ab |
| #2 | (proteom* OR "mass spectrometry" OR "mass-spectrometry" OR "mass spectrometric" OR "isobaric tags for relative and absolute quantification" OR iTRAQ OR "Tandem Mass Tag").ti |
| #3 | Proteome.mh. |
| #4 | Proteomics.mh. |
| #5 | Mass Spectrometry.mh. |
| #6 | Electrophoresis, Gel, Two-Dimensional.mh. |
| #7 | 1 OR 2 OR 3 OR 4 OR 5 OR 6 |
| #8 | (depress*).ab |
| #9 | (depress*).ti |
| #10 | depressive disorder.mh. |
| #11 | 8 or 9 or 10 |
| #12 | 7 and 11 |

| **Table S2. Numbers of potential citations for proteomics studies.** | | |
| --- | --- | --- |
| **Databases** | **URL** | **Citations** |
| PubMed | http://www.ncbi.nlm.nih.gov/pubmed | 3,160 |
| Web of Science | http://www.webofknowledge.com | 4,352 |
| Embase | http://www.embase.com | 5,801 |
| PsychInfo | https://www.ebscohost.com/nursing/products/psycinfo | 429 |
| **Total** |  | **13,742** |

| **Table S3. Numbers of full-text articles excluded.** | | |
| --- | --- | --- |
| **Excluded reasons** | **No. of excluded articles** | |
|  | **Proteomics data** | **Metabolomics data** |
| No experimental technique of interest | 40 | 1,057 |
| No study subject of interest | 57 | 406 |
| Other types of reports (review, case report, protocol, commentary, editorial) | 22 | 95 |
| Duplicate reporting | 12 | 79 |
| No control of interest | 14 | 57 |
| No differential analysis | 6 | 52 |
| Meeting abstracts | 5 | 28 |
| No relevant study | 45 | 37 |
| Others | 8 | 33 |
| Drug composition analysis | 0 | 96 |
| Pharmacokinetic study | 0 | 58 |
| No available data | 0 | 32 |
| Intervention study | 0 | 22 |
| **Total** | **209** | **2,052** |

| **Table S4. An example of the standardized data abstraction spreadsheet.** | | |
| --- | --- | --- |
| Franzen AD 2020 |  | |
| **Study ID** | Study P145 | |
| **Title** | Cerebrospinal fluid proteome evaluation in major depressive disorder by mass spectrometry | |
| **Overall design** | In this study, cerebrospinal fluid (CSF) samples were obtained from 15 major depressive disorder patients (MDD group), seven with major depressive disorder and eight age- and gender-matched non-psychiatric controls (control group). CSF protein profiles were obtained using label free-based quantitative mass spectrometry. Proteins with a p-value < 0.05 (ANOVA and Benjamini-Hochberg adjusted p values), were considered diﬀerentially expressed. | |
| **Study type** | Type (1) Depressed vs. non-depressed individuals | |
| **Organism** | Human | |
| **Categories of depression** | Major depressive disorder | |
| **Criteria for depression** | DSM-IV diagnosed MDD, HAMD-17 > 17 | |
| **Sample** | Cerebrospinal fluid | |
| **Sample size** | 15 | |
| **Platforms** | Label free | |
| **Data available** | Not available | |
| **Citation(s)** | Franzen AD, Lam TT, Williams KR, et al. Cerebrospinal fluid proteome evaluation in major depressive disorder by mass spectrometry. BMC Psychiatry. 2020;20(1):481. | |
| **PMID** | 32998701 | |
| **DOI** | 10.1186/s12888-020-02874-9 | |
| **Type (1) Depressed vs. non-depressed individuals** | | |
| **DE proteins (up)** | **UniProt accession** | **Gene symbol** |
| Cerebrospinal fluid  (MDD group vs. control group) |  |  |
|  | P01011 | AACT_HUMAN |
|  | P16035 | TIMP2_HUMAN |
|  | P00450 | CERU_HUMAN |
|  | P02746 | C1QB_HUMAN |
|  | P02750 | A2GL_HUMAN |
|  | Q8TAG5 | VTM2A_HUMAN |
|  | P02671 | FIBA_HUMAN |
|  | P36222 | CH3L1_HUMAN |
|  | Q9P2S2 | NRX2A_HUMAN |
|  | A0A0A6YYD4 | TVB13_HUMAN |
|  | P16070 | CD44_HUMAN |
|  | Q13094 | LCP2_HUMAN |
|  | P02675 | FIBB_HUMAN |
|  | P51884 | LUM_HUMAN |
|  | P00746 | CFAD_HUMAN |
|  | P01009 | A1AT_HUMAN |
|  | P02679 | FIBG_HUMAN |
|  | P0DOX6 | IGM_HUMAN |
|  | Q8IVL0 | NAV3_HUMAN |
|  | O75460 | ERN1_HUMAN |
| **DE proteins (down)** | **UniProt accession** | **Gene symbol** |
| Cerebrospinal fluid  (MDD group vs. control group) |  |  |
|  | P29622 | KAIN_HUMAN |
|  | P60174 | TPIS_HUMAN |
|  | O43293 | DAPK3_HUMAN |
|  | A0A075B6K4 | LV310_HUMAN |
|  | P04090 | REL2_HUMAN |
|  | Q8IVW6 | ARI3B_HUMAN |
|  | P0C0L5 | CO4B_HUMAN |
|  | Q6P1S2 | CC033_HUMAN |
|  | P25705 | ATPA_HUMAN |
|  | P02765 | FETUA_HUMAN |
|  | Q8TBN0 | R3GEF_HUMAN |
|  | Q13428 | TCOF_HUMAN |
|  | P08697 | A2AP_HUMAN |
|  | P01008 | ANT3_HUMAN |
|  | Q9NQV8 | PRDM8_HUMAN |
|  | P19652 | A1AG2_HUMAN |
|  | P0DPD6 | ECE2_HUMAN |
|  | P04004 | VTNC_HUMAN |
|  | P07339 | CATD_HUMAN |

| **Table S5. Consistently altered molecules identified by vote-counting procedure.** | | | | | |
| --- | --- | --- | --- | --- | --- |
| **Molecules** | **No. of studies that report on the molecules** | | | **Vote-counting statistic** | ***P* value** |
|  | **All** | **Upregulated** | **Downregulated** |  |  |
| L-Kynurenine | 38 | 36 | 2 | 34 | <0.001 |
| Hydroxykynurenine | 21 | 20 | 1 | 19 | <0.001 |
| Kynurenine/Tryptophan ratio | 18 | 18 | 0 | 18 | <0.001 |
| myo-Inositol | 36 | 27 | 9 | 18 | 0.002 |
| Glycerophosphocholine | 17 | 14 | 3 | 11 | 0.006 |
| 5-HIAA/5-HT ratio | 11 | 10 | 1 | 9 | 0.006 |
| Hint1 | 8 | 8 | 0 | 8 | 0.004 |
| Ndufs6 | 8 | 8 | 0 | 8 | 0.004 |
| Quinolinic acid | 10 | 9 | 1 | 8 | 0.011 |
| Mdh1 | 12 | 10 | 2 | 8 | 0.019 |
| myo-Inositol/(Creatine and Phosphocreatine) ratio | 7 | 7 | 0 | 7 | 0.008 |
| Atp4a | 7 | 7 | 0 | 7 | 0.008 |
| Ldhb | 7 | 7 | 0 | 7 | 0.008 |
| Hpx | 9 | 8 | 1 | 7 | 0.020 |
| C3 | 6 | 6 | 0 | 6 | 0.016 |
| Cox6a1 | 8 | 7 | 1 | 6 | 0.035 |
| Ndrg2 | 8 | 7 | 1 | 6 | 0.035 |
| Cp | 5 | 5 | 0 | 5 | 0.031 |
| Cplx1 | 5 | 5 | 0 | 5 | 0.031 |
| Gtf2i | 5 | 5 | 0 | 5 | 0.031 |
| Orm1 | 5 | 5 | 0 | 5 | 0.031 |
| S100b | 5 | 5 | 0 | 5 | 0.031 |
| Syt15 | 5 | 5 | 0 | 5 | 0.031 |
| Tgm2 | 5 | 5 | 0 | 5 | 0.031 |
| Tuba1c | 5 | 5 | 0 | 5 | 0.031 |
| Ywhah | 5 | 5 | 0 | 5 | 0.031 |
| (Glutamate and Glutamine)/(Creatine and Phosphocreatine) ratio | 5 | 0 | 5 | −5 | 0.031 |
| Creatinine | 5 | 0 | 5 | −5 | 0.031 |
| Epinephrine | 5 | 0 | 5 | −5 | 0.031 |
| Adi1 | 5 | 0 | 5 | −5 | 0.031 |
| Atp6v0a2 | 5 | 0 | 5 | −5 | 0.031 |
| Basp1 | 5 | 0 | 5 | −5 | 0.031 |
| Pllp | 5 | 0 | 5 | −5 | 0.031 |
| Eicosapentaenoic acid | 6 | 0 | 6 | −6 | 0.016 |
| Ndufv2 | 6 | 0 | 6 | −6 | 0.016 |
| Pacc1 | 6 | 0 | 6 | −6 | 0.016 |
| Psmb4 | 6 | 0 | 6 | −6 | 0.016 |
| Serotonin/Tryptophan ratio | 8 | 1 | 7 | −6 | 0.035 |
| Atp6v0c | 8 | 1 | 7 | −6 | 0.035 |
| Guanosine | 7 | 0 | 7 | −7 | 0.008 |
| Pantothenic acid | 7 | 0 | 7 | −7 | 0.008 |
| N-Acetyl aspartate/(Creatine and Phosphocreatine) ratio | 9 | 1 | 8 | −7 | 0.020 |
| Prdx2 | 11 | 2 | 9 | −7 | 0.033 |
| Niacinamide | 10 | 1 | 9 | −8 | 0.011 |
| Calr | 10 | 1 | 9 | −8 | 0.011 |
| Citric acid | 14 | 3 | 11 | −8 | 0.029 |
| Kynurenic acid | 14 | 3 | 11 | −8 | 0.029 |
| Docosahexaenoic acid | 15 | 3 | 12 | −9 | 0.018 |
| Creatine | 22 | 6 | 16 | −10 | 0.026 |
| L-Tyrosine | 26 | 8 | 18 | −10 | 0.038 |
| 5-Hydroxyindoleacetic acid | 28 | 9 | 19 | −10 | 0.044 |
| L-Phenylalanine | 28 | 9 | 19 | −10 | 0.044 |
| Anandamide | 20 | 4 | 16 | −12 | 0.006 |
| L-Glutamine | 58 | 20 | 38 | −18 | 0.012 |
| N-Acetyl-L-aspartic acid | 53 | 17 | 36 | −19 | 0.006 |
| L-Tryptophan | 56 | 18 | 38 | −20 | 0.005 |
| Norepinephrine | 39 | 7 | 32 | −25 | <0.001 |
| Gamma-Aminobutyric acid | 71 | 16 | 55 | −39 | <0.001 |
| Dopamine | 54 | 5 | 49 | −44 | <0.001 |
| Serotonin | 88 | 9 | 79 | −70 | <0.001 |
| Abbreviations. 5-HIAA, 5-Hydroxyindoleacetic acid; 5-HT, serotonin. | | | | | |

Supplemental Figures

**Fig. S1** **An overview of included studies and molecular entries in human, non-human primate, rat, and mouse studies.** (**A**) The numbers of included studies in each organism. (**B**) The numbers of included studies in each tissue. Top 5 frequently used tissues are shown.


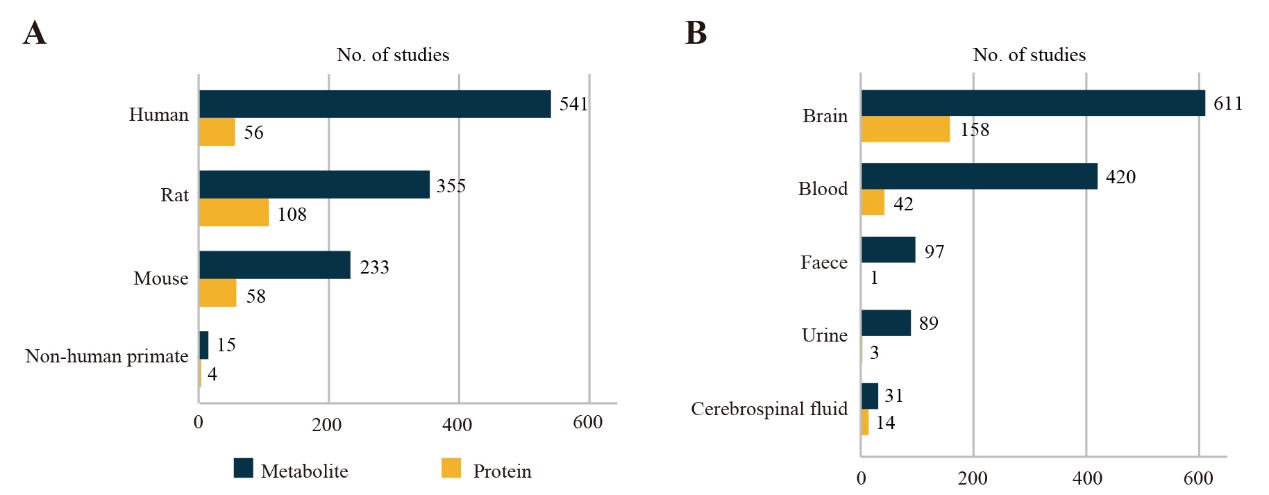


**Fig. S2 The number of entries for the most frequently reported molecules in human, non-human primate, rat, and mouse studies.** Top 10 frequently reported metabolites and proteins are shown.


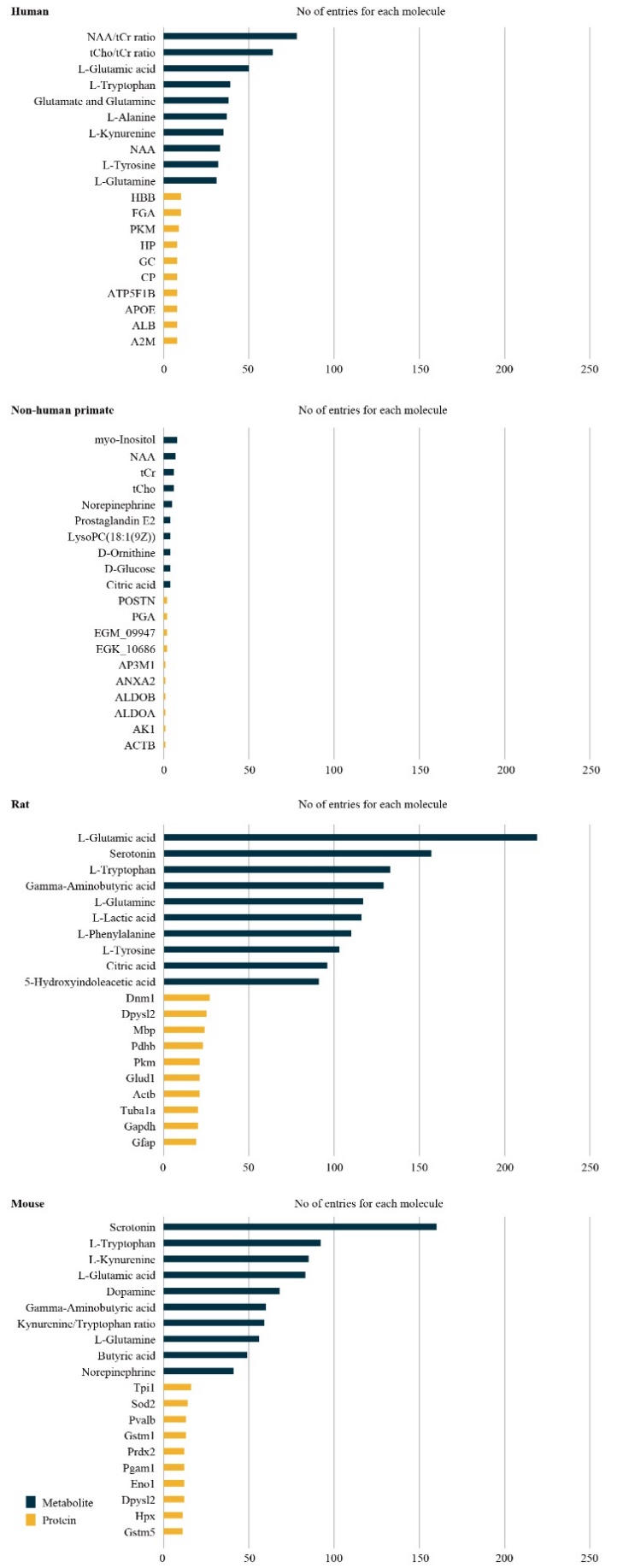

Supplement: Supplementary file 1 — Supplement [file 41398_2024_2948_MOESM1_ESM.docx]
